# 基于非靶向代谢组学的茄梨和红茄梨成熟期果皮代谢产物的差异分析

**DOI:** 10.3724/SP.J.1123.2021.05002

**Published:** 2021-11-08

**Authors:** Hongmei MU, Zhijuan CI, MAMAT Aisajan, Yanping LIANG, Xiaohong LIU, Xiaoyun DU, Qiang YU, Qingyu LI, Yuanjun LI

**Affiliations:** 1.山东省烟台市农业科学研究院, 山东 烟台 265500; 1. Yantai Academy of Agricultural Sciences Shandong Province, Yantai 26550, China; 2.新疆农业科学院, 新疆 乌鲁木齐 830091; 2. Xinjiang Academy of Agricultural Sciences, Urumchi 830091, China; 3.云南省农业科学研究院, 云南 昆明 650205; 3. Yunnan Academy of Agricultural Sciences, Yunnan 650205, China

**Keywords:** 超高效液相色谱-质谱, 植物代谢组学, 差异代谢物, 梨, 果皮, ultra performance liquid chromatography-tandem mass spectrometry (UPLC-MS/MS), plant metabolomics, differential metabolites, pear, fruit skin

## Abstract

为了探究西洋梨品种茄梨及其红色芽变红茄梨成熟期果皮代谢产物差异,采用超高效液相色谱-质谱联用技术,对茄梨和红茄梨成熟期果皮进行非靶向代谢组学研究。通过主成分分析和正交偏最小二乘判别分析,构建了多变量统计分析模型,结合模型和变量重要性投影与最大差异倍数值,基于精确质量数、二级碎片以及同位素分布,使用PMDB(Plant Metabolome Database)数据库进行定性,筛选并鉴定出茄梨和红茄梨果皮中显著性变化(*P*<0.05, VIP(variable importance in project)≥1)的差异代谢物有83种,主要包括酚酸类、黄酮类和氨基酸类物质,涉及类黄酮代谢、氨基酸代谢、苯丙烷类代谢等代谢途径,其中53种物质含量上调,30种物质表达下调。通过KEGG(Kyoto Encyclopedia of Genes and Genomes)数据库进一步对差异代谢物质进行通路富集分析,差异代谢物主要分布在20条代谢途径中,*P*<0.05的代谢途径有6条,分别是类黄酮生物合成、黄酮和黄酮醇生物合成、苯丙烷生物合成、丁酸酯代谢、苯丙氨酸代谢、酪氨酸代谢。这些差异代谢物的变化可能是导致茄梨和红茄梨果皮色泽不同的原因。该研究从植物代谢组学角度初步揭示了茄梨和红茄梨成熟期果皮的代谢产物差异性。

## Abstract (en)

Red Clapp’s Favorite is the red mutation cultivar of the pear cultivar Clapp’s Favorite. Fruit color is an important feature of pear fruits, with red skin generally attracting consumers. Anthocyanin, chlorophyll, and carotenoids are the most important pigments in the color formation of fruits. The red color of pear skin is mainly due to the concentration and composition of anthocyanin. Metabolomics is an emerging discipline that focuses on the qualitative and quantitative analysis of small metabolites with low molecular weight in biological cells and tissues. As an important part of systems biology, it is an effective means to solve many complex biological problems. Studies have analyzed pigment content, composition, and differentially expressed genes in the skin of green and red pears from various aspects. Anthocyanins are responsible for physiological activity on regulating pathways. The aim of this study was to discover differential metabolites in the skin of Clapp’s Favorite and its red mutation cultivar Red Clapp’s Favorite. The metabolic components were detected using high-performance liquid chromatography coupled with tandem mass spectrometry (UPLC-MS/MS). Chromatographic experiments were performed on an HSS T3 column (100 mm×2.1 mm, 1.8 μm) by using a mobile phase consisting of 0.1% (v/v) formic acid in acetonitrile and water, and mass spectrometry was conducted in the positive and negative modes by electrospray ionization (ESI). Red Clapp’s Favorite and Clapp’s Favorite were collected from the pear germplasm resource nursery of Yantai Institute of Agricultural Sciences in Shandong. The data were analyzed by principal component analysis (PCA) and orthogonal partial least squares discriminant analysis (OPLS-DA) as well as cluster analysis and heat map. The first two principal components exhibited 62.3% and 8% of the total variance in the positive and negative ion modes, respectively. PCA can generally reflect metabolite differences between the two groups of samples, and there are significant differences in metabolites between the two cultivars. The results showed that PLS-DA clearly distinguishes the two groups of samples, which can be used to analyze the subsequent difference in components. The compounds were identified based on data retrieved from the PMDB databases according to the accurate mass number, secondary fragment, and isotope distribution. The results showed that the metabolite content in the skin of Red Clapp’s Favorite and Clapp’s Favorite were significant. There were 83 different metabolites (*P*<0.05, variable importance in project (VIP)≥1), including phenols and amino acids, which are involved in flavonoid metabolism, amino acid metabolism, phenyl propanoid biosynthesis, and other metabolic pathways, including 5 polyphenols, 3 flavonoids, 1 amino acid and derivatives, 8 phenylpropanes, 2 anthocyanins, 5 proanthocyanidins, 6 flavanols, 14 flavonols, 2 isoflavones, 13 triterpenoids, 3 organic acids and derivatives, 1 vitamin, 3 organic acids and derivatives, 15 lipids, and 2 other compounds. The chlorogenic acid and crypto-chlorogenic acid in Red Clapp’s Favorite are 2.40 and 3.46 times as much as those in Clapp’s Favorite. The anthocyanins of cornulin 3-glucoside and cornulin 3-galactoside were 10.235 and 9.394 times, respectively. Phenolic epicatechin and catechin increased by 4.689 and 4.635, respectively. The content of phenylpropane 3, 4-dihydroxycinnamic acid in Red Clapp’s Favorite increased by 3.13 times. Among the 83 differential metabolites, 23 metabolites were enriched in the pathway. To display the relationship between the samples and the differences in metabolites among the different samples intuitively, hierarchical clustering and heat map analysis were performed on the metabolite expression levels with significant differences in the enrichment pathways. The Kyoto Encyclopedia of Genes and Genomes database was used to further analyze the pathway enrichment of different metabolites. According to the results, there were 6 metabolic pathways (*P*<0.05): flavonoid biosynthesis, flavone and flavonol biosynthesis, phenylpropanoid biosynthesis, butanoate metabolism, phenylalanine metabolism, and tyrosine metabolism. Plant secondary metabolism shows a complex diversity. This study would screen out other pathways affecting the biosynthesis of flavonoids, which could provide reference for the further study of biosynthesis and biological function of flavonoids in red fruits. This study provides a useful reference for metabolomics of red pears, which could provide a theoretical reference for the quality analysis and biological function research of pears.

梨是蔷薇科梨属(Pyrus L.)植物,按起源分为东方梨和西方梨两大种群^[1]^。茄梨原产美国,在我国胶东地区栽培较多,果皮黄绿色,果实肉质细腻,软溶多汁。红茄梨为茄梨的芽变品种,其果皮呈深红色。果皮色泽是果实重要的外观品质性状,是果实品质和商品性的重要组成部分,红色果实不仅外观漂亮,还富含抗氧化作用的功能色素成分,具有延缓衰老、防治心血管和慢性疾病等多种功效^[2]^。现有研究已从多方面对绿皮梨和红皮梨果皮色素含量、组成以及差异表达基因等进行了分析,认为果皮红色主要是由于花青苷含量的不同引起的^[3]^。

代谢组学是一门新兴学科,是对生物体细胞、组织中非常小的低分子量代谢产物进行定性和定量分析的研究^[4]^,作为系统生物学的重要部分,是解决诸多复杂生物学问题的有效手段之一^[5,6]^。其中,非靶向代谢组学能从整体反映代谢物的变化,有利于发现新的代谢通路等^[7]^。近年来,基于液相色谱-质谱技术的代谢组学分析在品种鉴定、营养科学等方面^[8]^广泛应用,且具有其他检测手段无法比拟的优势^[9]^。超高效液相色谱-串联高分辨质谱法分辨率高,精准分子质量,具有同时实现母核、子离子的高分辨采集及质谱碎裂信息获取等优点^[10]^。孙莉琼等^[11]^采用超高效液相色谱-串联质谱(UPLC-MS/MS)的多离子反应监测模式,开展代谢组学研究,初步确定了梨属果实的特征性多酚和三萜酸类物质。李静等^[12]^采用超高效液相色谱-二极管阵列-串联四极杆质谱联用方法鉴别砀山酥梨和秋白梨的酚类组成的差异。李浩男等^[13]^采用GC-MS和LC-MS方法分析黄花及其芽变绿黄花梨果皮中代谢物,发现二者果皮色泽上的差异是由差异代谢产物苯丙烷类化合物、脂肪酸类化合物以及酚类化合物等共同作用的结果。运用代谢组学方法分析不同颜色西洋梨果皮中代谢物质尚未见报道。

本研究采用UPLC-MS技术,以茄梨和红茄梨果皮作为研究材料,利用非靶向代谢组学方法进行代谢组学分析,运用多元统计方法对数据进行模式识别,并检索相关数据库进行差异代谢物的结构推测,从植物代谢组学角度初步揭示茄梨和红茄梨果皮差异代谢产物,对于更为深入地研究红茄梨品质形成的分子机理有积极的作用。

## 1 实验部分

### 1.1 仪器、试剂与材料

AB ExionLC高效液相色谱仪和AB TripleTOF 6600 plus高分辨质谱仪(AB Sciex);高分辨质谱仪全自动样品快速研磨仪(上海净信实业发展有限公司); F-060SD超声波清洗机(深圳福洋科技集团有限公司); TYXH-I漩涡振荡器(上海汗诺仪器有限公司); TGL-16MS台式高速冷冻离心机(上海芦湘仪离心机仪器有限公司); LNG-T98冷冻浓缩离心干燥器(太仓市华美生化仪器厂)。

甲醇、甲酸、水、乙腈均购自Thermo公司,L-2-氯苯丙氨酸购自上海恒创生物科技有限公司。所有化学药品和溶剂均为分析纯或色谱级。

### 1.2 实验条件

1.2.1 样品采集和制备

2020年7月28日,在山东省烟台市农业科学研究院梨种质资源圃的3株10年生茄梨和红茄梨树上,分别采集茄梨和红茄梨各15个成熟期果实,放入冰盒,带回实验室,待后熟用手术刀分离0.1~0.2 cm厚的果皮混合,液氮速冻后保存在-80 ℃的冰箱备用,重复6次。将上述样品称取80 mg,加入20 μL内标(0.3 mg/mL L-2-氯苯丙氨酸的甲醇溶液)和1 mL的甲醇-水(7:3, v/v); -20 ℃下预冷2 min后冰水浴超声提取30 min, -20 ℃过夜静置;13000 r/m、4 ℃下离心10 min,用注射器吸取150 μL的上清液,使用0.22 μm的有机相针孔过滤器过滤后转移到LC进样小瓶中,-80 ℃下保存。

1.2.2 色谱条件

色谱柱:ACQUITY UPLC HSS T3(100 mm×2.1 mm, 1.8 μm);注温:45 ℃,流速:0.35 mL/min;流动相A是水(含0.1%甲酸), B是乙腈(含0.1%甲酸);按照体积比进行梯度洗脱,洗脱梯度为:0~4 min, 5%B~30%B; 8~10 min, 50%B~80%B; 14~15 min, 100%B; 15.1~16 min, 5%B。进样体积:2 μL。

1.2.3 质谱条件

分别在ESI正、负离子模式下进行扫描,离子喷雾电压分别为5500和4500 V,离子源温度550 ℃;全扫描模式(*m/z* 40~1000)结合IDA(information dependent acquisition)模式,二级质量扫描范围*m/z* 100~1000。

1.2.4 数据分析

采用组学数据处理软件Progenesis QI v2.3对原始数据进行定性及相对定量分析,并对原始数据进行标准化预处理。所有峰信号强度(峰面积)筛选后分段归一化处理,进行峰识别、峰过滤等,对同一代谢物在不同样本中的质谱出峰图进行校正^[14]^。采用主成分分析(PCA)、正交偏最小二乘判别分析(OPLS-DA)等多元统计分析区分各组间代谢的总体差异,筛选差异代谢物。基于精确质量数、二级碎片以及同位素分布,使用PMDB(Plant Metabolome Database)数据库推测化合物结构。利用差异代谢物的KEGG(Kyoto Encyclopedia of Genes and Genomes)进行通路富集分析,获得代谢通路富集结果。应用超几何检验,找出与整个背景相比在显著性差异表达代谢物中显著富集的代谢通路条目,其计算公式为:

*P*=1- $\overset{m-1}{\underset{i=0}{\sum }}\frac{\left(\frac{M}{i}\right)\left(\frac{N-M}{n-i}\right)}{\left(\frac{N}{n}\right)}$

其中,*N*为代谢物总数;*n*为*N*中差异表达代谢物的数目;*M*为注释为某特定代谢通路的代谢物数目;*m*为注释为某特定代谢通路的差异代谢物数目。以*P*≤0.05为阈值,满足此条件的代谢通路为在差异代谢物中显著富集的代谢通路。*P*越小,则该代谢通路的差异越显著。

## 2 结果与讨论

### 2.1 色谱-质谱分析结果

正、负离子模式下茄梨和红茄梨的总离子流色谱图如图1所示,2个品种整体轮廓存在一定差异,为了揭示茄梨和红茄梨间的组分差异,进一步采用多维模式识别,对反映样本的多个变量进行观测,以进行较为全面的分析。

**图1:**
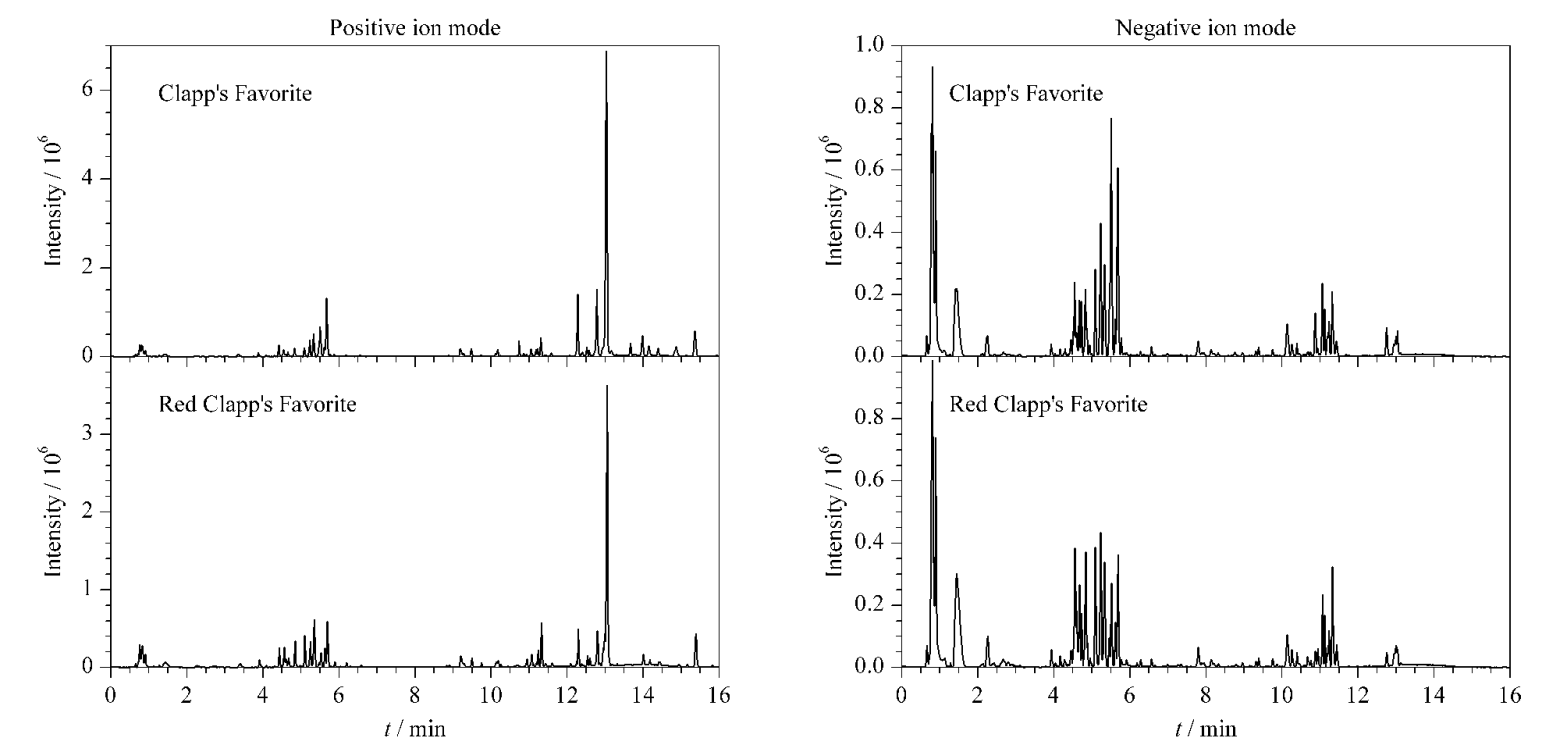
茄梨和红茄梨在正、负离子模式下的总离子流色谱图

### 2.2 茄梨和红茄梨的代谢判别分析

2.2.1 主成分分析

通过多元统计PCA对样品进行分析。3个主成分中,主成分1的贡献率为62.3%,主成分2的贡献率为8.0%。*X*轴方向模型的累积解释率*R*^2^*X*=0.751,因此该PCA模型拟合性较好。PCA得分图见图2。红茄梨和茄梨组间样品明显聚为2组,基本得到了有效区分,说明组间存在显著差异。

**图2:**
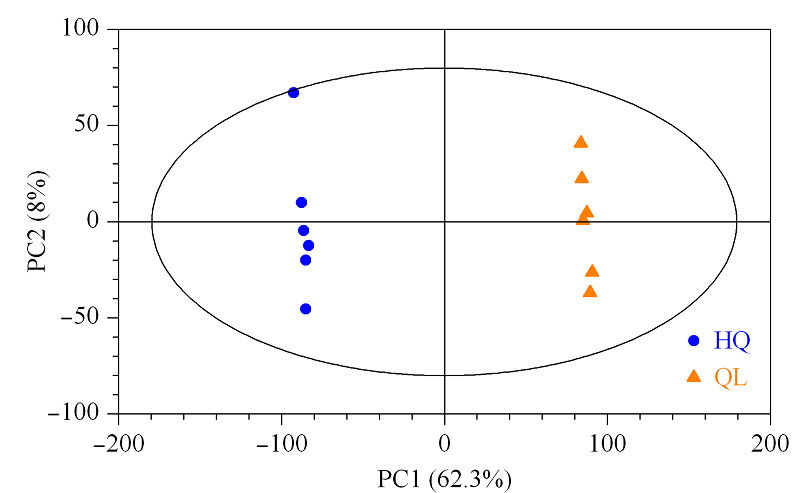
茄梨和红茄梨的PCA得分图

2.2.2 正交偏最小二乘判别分析

OPLS-DA可以过滤掉与分类无关的信息,准确分析组间差异^[15]^。采用OPLS-DA对得到的质谱数据进行分析,发现茄梨和红茄梨果皮样品分布在置信区间的左侧和右侧,区分效果明显,表明2组之间具有显著的差异(见图3)。其模型质量参数为3个主成分,模型累计预测率*Q*^2^=0.998, *R*^2^*X*=0.878,图中*Q*^2^<0,且所有*Q*^2^点从左到右始终低于原始蓝色的*Q*^2^点,说明该评估模型可靠有效。

**图3:**
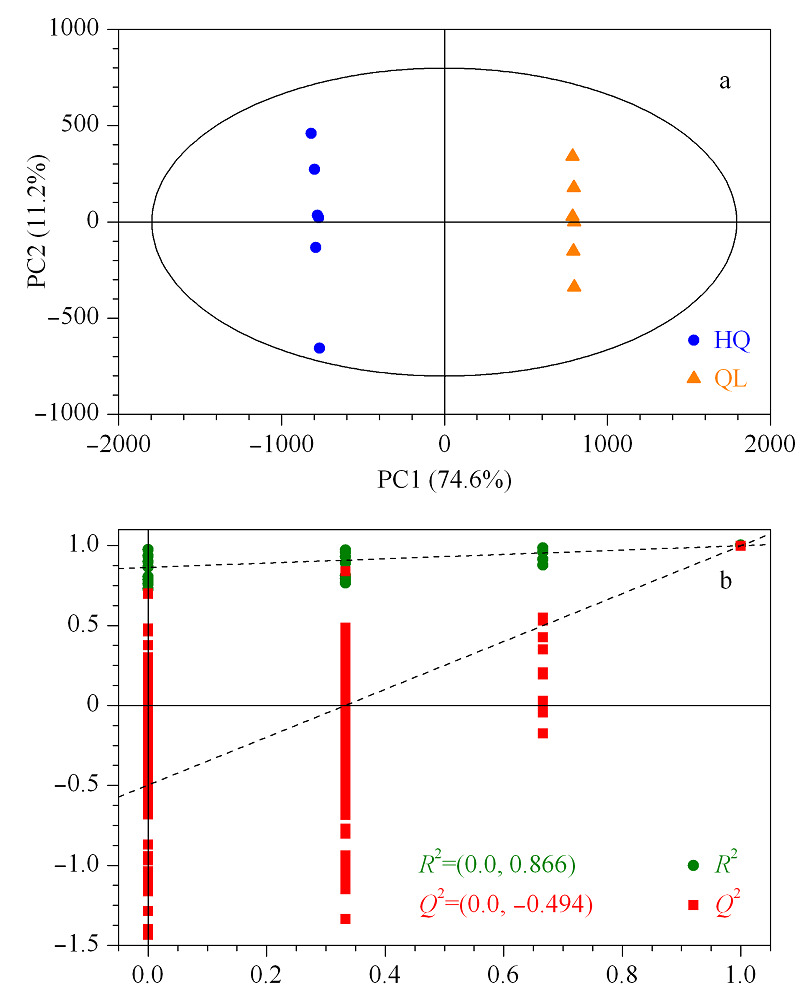
茄梨和红茄的(a)OPLS-DA得分图和(b)置换检验图

### 2.3 差异代谢物的筛选与鉴别

基于前体离子的精准质量数、前体离子同位素组成以及碎片离子的信息进行元素组成的确定,在PMDB等数据库中对鉴定的化合物进行前体离子与碎片离子的匹配评分,鉴定差异代谢物。根据变量重要性投影^[16]^(variable importance in project(VIP)≥1, *P*<0.05)和最大差异倍数值,筛选出83种差异显著代谢物,包括酚类5种、黄酮类3种、氨基酸及衍生物1种、苯丙烷类8种,花色苷类2种,原花青素5种,黄烷醇6种,黄酮醇14种,异黄酮2种,三萜类13种,有机酸及衍生物3种,维生素1种,有机氧化物3种,脂类15种,其他类2种(见表1)。其中,上调物质有53种,占63.86%;下调物质有30种,占36.14%。

**表1:** 红茄梨与茄梨果皮中的差异性代谢产物

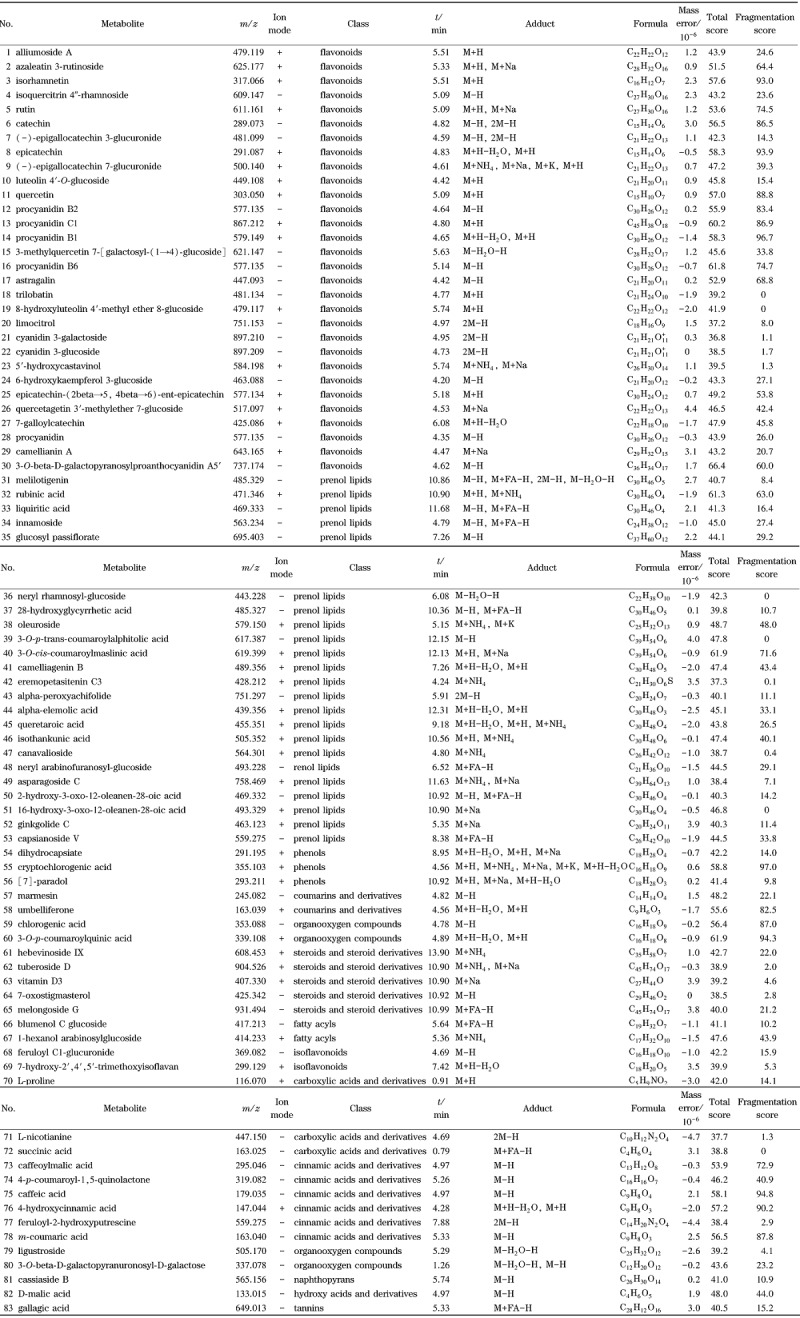

研究发现红茄梨中含有种类丰富的黄酮类化合物,红茄梨中矢车菊素3-葡萄糖苷、矢车菊素3-半乳糖苷是茄梨的9.39和10.24倍,推测矢车菊素3-半乳糖苷和矢车菊素3-葡萄糖苷是红茄梨红色的主要因素。其他类黄酮组分,特别是儿茶素、表儿茶素等黄烷醇类物质,槲皮素(黄酮醇)和芦丁、木犀草素(黄酮类)等含量均与茄梨有较大差异,可能其他类黄酮组分的变化也影响了果皮颜色的变化。黄酮醇合成酶(FLS)和二氢黄酮醇-4-还原酶(DFR)会通过底物竞争影响花青素苷和黄酮醇的比例,进而使颜色发生改变。红茄梨中槲皮素、芦丁含量上调,木犀草素和异属李素含量下调,可能是红茄梨中类黄酮代谢途径中相关调控因素的影响,也可能是黄酮类化合物与花青素存在底物竞争性。

儿茶素具有抗氧化、保护心脑血管、抗肿瘤等作用,没食子酸具有抗氧化和抗血栓等药理作用,是次生物质代谢的重要成分。本试验中检测到的红茄梨果皮中酚类物质表儿茶素、儿茶素比茄梨中的含量明显升高。原花青素类物质原花青素B2、原花青素C1、原花青素B1、原花青素B6分别是茄梨的4.42、4.62、4.47、3.86倍。红茄梨中原花青素和类黄酮糖苷类衍生物维持在较高水平,意味着红茄梨除具有外观鲜艳的特征外,还具备一定保健或药用潜力。

S图见图4,图中可视化的所有点全部分布在第一和第三象限,类似S形,越靠近S形末尾两端,贡献越大,差异越显著^[17]^。为了更直观地展示样本之间的关系及代谢物在不同样本之间的表达差异,我们对富集在通路上的显著差异代谢物表达量进行了热图分析(见图5)。蓝色为低表达物质,红色为高表达代谢物。QL和HQ组明显分开,说明两组之间存在明显差异,且筛选的显著差异代谢物能作为标志物将两组区分开。聚在一簇的代谢物具有相似功能或共同参与同一代谢途径。

**图4:**
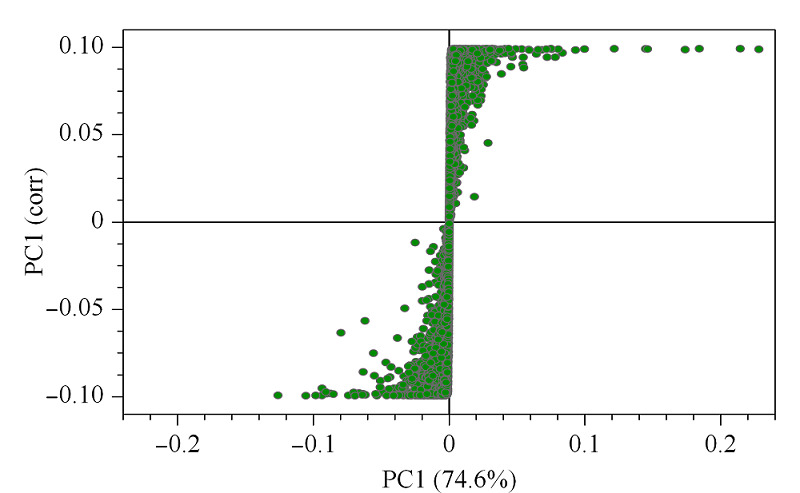
正交偏最小二乘判别分析的S图

**图5:**
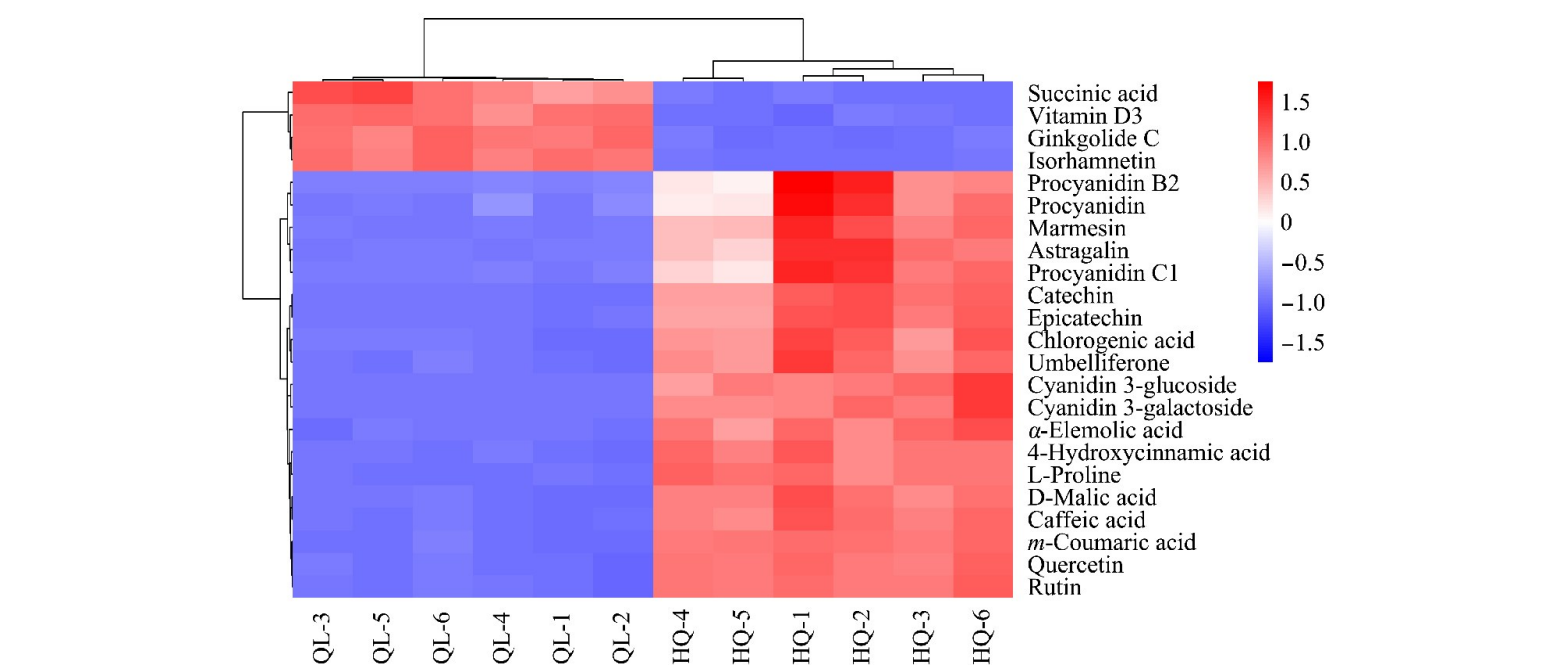
茄梨和红茄梨果皮中富集在通路上的差异代谢物热图

### 2.4 差异代谢物通路分析

通过KEGG数据库进一步对有差异的代谢物质进行通路富集分析^[18]^。差异代谢物主要分布在20条代谢途径中,*P*<0.05的代谢途径有6条,分别是:类黄酮生物合成(flavonoid biosynthesis);黄酮和黄酮醇生物合成(flavone and flavonol biosynthesis);苯丙烷生物合成(phenylpropanoid biosynthesis);丁酸酯代谢(butanoate metabolism);苯丙氨酸代谢(phenylalanine metabolism);酪氨酸代谢(tyrosine metabolism)(见图6)。其中,类黄酮生物合成代谢通路最为显著,*P*值为0.0001317, -log *P*为3.88,主要有4个被检测到的差异代谢物富集于此代谢通路上,分别为槲皮素、绿原酸、儿茶素和表儿茶素,与黄酮类化合物的合成和花色苷的积累密切相关。黄酮醇是类黄酮合成途径的支路之一,黄酮醇合成酶(FLS)是黄酮类化合物合成途径与儿茶素合成途径的桥梁,二氢槲皮素在FLS作用下生成槲皮素,在二氢黄酮醇4-还原酶(DFR)作用下生成无色矢车菊素,在DFR作用下,被还原成无色花青素苷^[19]^。

**图6:**
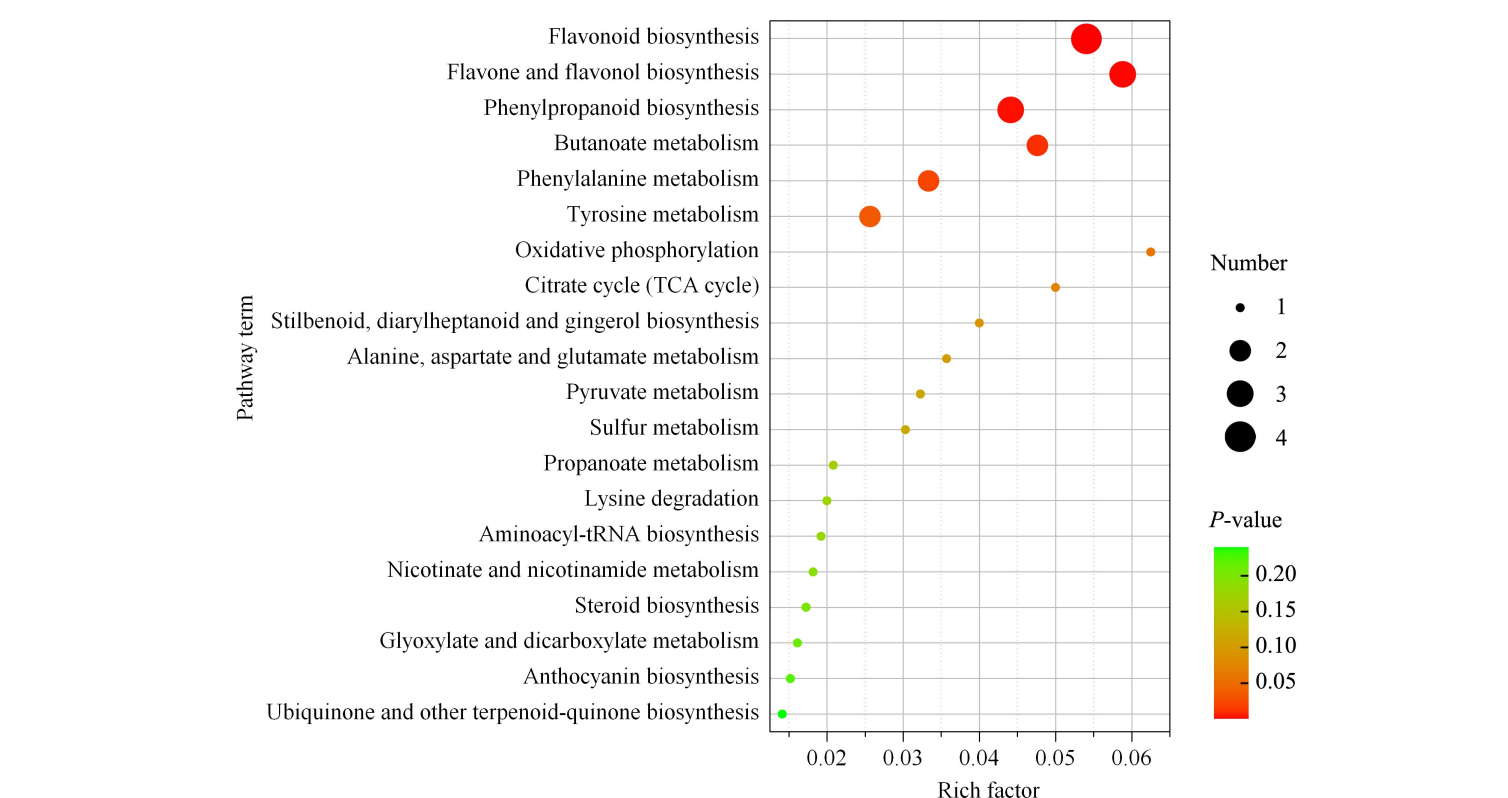
茄梨和红茄梨果皮中差异代谢产物的KEGG富集图

富集在丁酸酯代谢途径上物质有2种,其中,丁二酸含量下调,D-苹果酸含量上调。4-羟基肉桂酸和间香豆酸富集在苯丙烷生物合成途径、苯丙氨酸代谢和酪氨酸代谢途径上,且含量上调,红茄梨可能是通过苯丙烷类物质的上调来促进酚类物质的积累,提高其抗氧化性,说明红茄梨通过相关物质含量的上调及相关通路的调节来影响果皮颜色的变化。

## 3 结论

植物次生代谢呈现出复杂的多样性,本文建立了基于超高效液相色谱-质谱联用对茄梨和红茄梨果皮差异次生代谢产物的非靶向代谢组学分析方法。方法快速可靠,具有稳定性高、快速准确、操作简便、重现性好等优点,该模型可以将茄梨和红茄梨彼此区分。本研究对茄梨和红茄梨果皮代谢产物进行初步比较,所检测到的物质可为梨果实品质分析及相应的生物学功能研究提供一定的理论参考。
